# A New Random Forest Algorithm Based on Learning Automata

**DOI:** 10.1155/2021/5572781

**Published:** 2021-03-27

**Authors:** Mohammad Savargiv, Behrooz Masoumi, Mohammad Reza Keyvanpour

**Affiliations:** ^1^Faculty of Computer and Information Technology Engineering, Qazvin Branch, Islamic Azad University, Qazvin, Iran; ^2^Department of Computer Engineering, Alzahra University, Tehran, Iran

## Abstract

The goal of aggregating the base classifiers is to achieve an aggregated classifier that has a higher resolution than individual classifiers. Random forest is one of the types of ensemble learning methods that have been considered more than other ensemble learning methods due to its simple structure, ease of understanding, as well as higher efficiency than similar methods. The ability and efficiency of classical methods are always influenced by the data. The capabilities of independence from the data domain, and the ability to adapt to problem space conditions, are the most challenging issues about the different types of classifiers. In this paper, a method based on learning automata is presented, through which the adaptive capabilities of the problem space, as well as the independence of the data domain, are added to the random forest to increase its efficiency. Using the idea of reinforcement learning in the random forest has made it possible to address issues with data that have a dynamic behaviour. Dynamic behaviour refers to the variability in the behaviour of a data sample in different domains. Therefore, to evaluate the proposed method, and to create an environment with dynamic behaviour, different domains of data have been considered. In the proposed method, the idea is added to the random forest using learning automata. The reason for this choice is the simple structure of the learning automata and the compatibility of the learning automata with the problem space. The evaluation results confirm the improvement of random forest efficiency.

## 1. Introduction

Random forest is one of the methods of ensemble learning that comes under the homogeneous base learner category in terms of the type of constructive classifiers. As the name implies, all base learners are decision trees, and therefore they have a simpler structure than similar methods [1]. The random forest structure has two advantages. The first category is from a computational point of view, and the second category is from a statistical point of view. Advantages that can be considered from a computational point of view are: the random forest has the ability to deal with both regression and classification issues. The train and prediction processes in this classifier are performed at high speed, and therefore the random forest is known as one of the fast classic classifiers. Another advantage of the random forest is its ability to be used directly in high-dimensional issues [2]. The advantages of the second view of the random forest are its characteristics, namely, prioritization of features, attribution of different weight coefficients to different classes, and illustration and unsupervised learning ability.

According to the literature, the random forest method is one of the most practical methods of ensemble learning. Weighting the base learners in ensemble learning is one of the main challenges in aggregating the basic classifiers in order to achieve a stronger classifier [3]. The reason for weighing base learners, or in other words, determining the impact factor for each base learner, is to increase the scalability of the data mining algorithm with the problem space. This becomes even more apparent when the environment is dynamic, and different or sometimes contradictory behaviours are observed from data in different situations. The text data environment has such an interesting behaviour that it challenges data mining algorithms. For example, placing one word on one domain may create a positive polarity, but it may also create a negative polarity on another domain. This difference in polarity is created without any change in the form of the word and without any change in the role of the word from a grammatical point of view. The word “small” in both the electronic domain and the restaurant domain has such a behaviour. This behaviour poses a major challenge to the opinion mining algorithms [4].

The classical solution in the literature to overcome this challenge is based on the use of lexical-based approaches. This approach is based on frameworks such as unigram, n-gram, aspect-based, and similar methods, and all of them are data-dependent. In addition to the urgent need for predefined data, these methods lose their efficiency if they are met with an unspecified word or metaphor in the opinion mining field. In other words, they are not compatible with the problem space. The way random forest works is that with the sequential placement of training data and feature vectors that are injected into each of the base learners, it tries to find the best subset of features, and by increasing their impact factor in the classifier, it achieves the highest performance among all the aggregated base learners [5]. However, this method is not effective in relation to data such as text, in which a word can have different polarities in different domains because, in the classification algorithm, there is no ability to adapt to the conditions of the problem space.

In this paper, we intend to empower random forest with the idea of reinforcement learning and improve its efficiency. In the proposed method, learning automata is used to aggregate and weigh base learners. The way learning automata works is to receive feedback from the environment and perform one of the actions based on the type of feedback. In the learning automata, feedbacks are divided into two categories of reinforcement signals: reward signals and penalty signals. For each reinforcement signal received by the learning automata, it updates the probability of selecting the selected action in the previous step. This process continues until the probability of action selections converges to one of the actions; in other words, the best option for running in the current situation is found. In the proposed method, learning automata actions are appropriate when one of the base learners selected leads to the maximum reward that can be received from the environment. Since at each stage of learning automata execution, the learning algorithm tries to select the best option, achieving global optima in the problem space is guaranteed. This is proof of the adaptability of the proposed method. In the proposed method, the subprocess of replacing features in the feature vector is removed, and all the features in the feature vector are used. As a practical application in the field of opinion mining, if the Bag of Word (BoW) method is used to create the feature vector, the advantage of considering all the features of the feature vector will also cover cases that occur rarely. In other words, in the proposed method, the aspect of independence from the domain in the processes such as opinion mining is considered.

Our contribution is summarized as follows:

  In this paper, a brief review of random forest in terms of application scope is given.
  In this paper, a learning automata-based method is proposed to improve the random forest performance.
  The proposed method operates independently of the domain, and it is adaptable to the conditions of the problem space.

The rest of the paper is organized as follows. In Section 2, related work is introduced.  Section 3 presents the introduction to learning automata. The proposed method is explained in Section 4.  Section 5 includes evaluation. Discussion is given in Section 6, and finally, the conclusion and future work are described in Section 7.

## 2. Related Work

In this section, theories and literature on the subject of random forest are examined. The purpose of this section is to review the innovations that have been introduced around random forest in recent years.

Random forest is considered as one of the methods of ensemble learning in the homogeneous ensemble learning subgroup. In the random forest, each decision tree, or in other words, each base learner, has access to a random subset of feature vectors [6]. Therefore, the feature vector is defined as follows:

(1) $$\begin{array}{c} x=\left(x_{1},x_{2},...,x_{p}\right), \end{array}$$,

where *p* is the dimension property of the available vector for the base learner. The main goal is to find the prediction function as *f*(*x*) that predicts the *Y* parameter. The prediction function is defined as follows:

(2) $$\begin{array}{c} L\left(Y,f\left(x\right)\right), \end{array}$$

where *L* is known as the loss function, and the goal is to minimize the expected value of the loss. For regression applications and classification applications, squared error loss and zero-one loss are common choices, respectively. These two functions are defined as follows in equations (3) and (4), respectively.

(3) $$\begin{array}{c} L\left(Y,f\left(x\right)\right)=\left(Y-f{\left(x\right)}^{2}\right), \end{array}$$

(4) $$\begin{array}{c} L\left(Y,f\left(x\right)\right)=I\left(Y\neq f\left(x\right)\right)=\left\{\begin{array}{c} 0,\text{ if }Y=f\left(x\right),\\ 1,\text{ otherwise.} \end{array}\right. \end{array}$$

To create an ensemble, a set of base learners come together. If base learners are defined as follows:

(5) $$\begin{array}{c} h_{1}\left(x\right),h_{2}\left(x\right),\ldots ,h_{J}\left(x\right), \end{array}$$

for regression applications, the averaging will be based on equation (6), and for classification applications, the voting will be based on equation (7).

(6) $$\begin{array}{c} f\left(x\right)=\frac{1}{J}\sum_{j=1}^{J}h_{j}\left(x\right), \end{array}$$

(7) $$\begin{array}{c} f\left(x\right)=\arg\max\sum_{j=1}^{J}I\left(y=h_{j}\left(x\right)\right). \end{array}$$

The Random Forest pseudocode for classification applications is shown in Algorithm 1.

As can be seen in Algorithm 1, in the random forest, an attempt is made to find a subset of features using the various replacements of training data and features that maximize the efficiency and accuracy of the output. This set of features is used to identify a new instance.

The following is a brief review of the random forest subject literature. It should be noted that we intend to introduce the background of the subject, and this paper is not a review paper, and the presented review is a brief review and does not mention all the previous works undoubtedly. However, the authors have tried to refer to the latest and most authoritative research work published in the recent years.

### 2.1. Astronomy, Bioinformatics, and Economics fields

In the astronomy field, Markel and Bayless [7] use RF for the classification of type IA and core-collapse supernovae. Chen et al. [8] propose an approach to detect the potential signal photons by RF. In the bioinformatics, Pang et al. [9] propose a method to mitigate the computational complexity of RNA simulation software by a typical random forest. Darmawan et al. [10] propose an age estimation model in the bioinformatics field. In the economics field, Park et al. [11] propose two stages of short-term load forecasting by random forest and deep neural networks to reduce energy costs. 12 use a typical RF to solve the e-commerce product classification problem. Modeling consumer credit risk by RF is the main goal of [13]. 14 increase tree correlation by controlling the probability of placing splits along with strong predictors to deal with high-dimensional settings. Sikdar et al. [15] proposed a variable selection method based on RF to identify the key predictors of price change in amazon.

### 2.2. General and Global Problem fields

In the general field, Giffon et al. [16] use the mean of orthogonal matching pursuit algorithms for calculating the weights of the linear combination for producing a linear combination of trees with minimum training error. Combining RF and generalized linear mixed models is the main idea of [17] to model clustered and longitudinal binary outcomes. Mohapatra et al. [18] optimize the random forest by use of unequal weight voting strategy. Ji et al. [19] propose a hybrid model for crowd counting by a combination of convolutional neural networks (CNN) and deep regression forest. Santra et al. [20] propose a deterministic dropout to remove unimportant connections in NN by RF. Proposing the oblique RF without explicit regularization techniques by minimizing the structural risk is the main goal of [21]. Katuwal et al. [22] use an oblique hyperplane to split the data for increasing the accuracy of the trees and reduce the depth of RF. Probst et al. [23] tune the hyper-parameters to achieve higher performance to improve the RF. Kim et al. [24] propose a method for interpreting and simplifying a black-box model of a deep RF by quantifying the feature contributions and frequency of the fully trained deep RF. Jain et al. [25] purpose dynamic weighing scheme for RF using the correlation between decision tree and data samples. In the global problem field, Stafoggia et al. [26] estimate daily particulate matter for weather forecasting by RF. Modeling the global forest area by RF is the main target of [27]. Breidenbach and Saravi [28] present research on land-subsidence spatial modeling and its assessment. Analyzing the net ecosystem carbon exchange is the goal of [29]. Prediction about the global climate problem using the index quantization ability of random forest and the optimizing ability of PSO in the NN prediction model is the main purpose of [30]. Li et al. [31] solve the class imbalance by detecting serial case pairs.

### 2.3. Healthcare field

Diagnosis detection and prediction of obesity in patients by RF are the main goals of [32, 33], respectively. El-Sappagh et al. [34] use RF in the simple form for the detection of Alzheimer's disease progression. In [35], RF is introduced as one useful machine learning tool for healthcare domain, especially for COVID-19 modeling. Khedkar et al. [36] use Patients Electronic Health Records for predicting the heart failure risks by RF. Hane et al. [37] propose a model for prediction of the dissolution behaviour of a wide variety of oxide glasses. Subudhi et al. [38] propose a method by RF to detect the ischemic stroke by a sequence of MRI images. Javadi et al. [39] propose a method to predict the immunogenic peptides of intracellular parasites. Identifying the key risk factors associated with acute rejection in organ transplantation is the main propose of [40]. In Singh et al. [41], RF has been used as one of the classifiers to classify the covid-19 spread. Na et al. [42] propose an automatic walking mode change of the above-knee prosthesis. Clustering and predicting vital signs by RF is the goal of [43]. Zhu et al. [44] optimize the parameters of the random forest by improved fish Swarm algorithm for predicting the knee contact force. A method for identifying foreign particles for quality detection of liquid pharmaceutical products is presented by [45]. Lee and Jung [46] consider the relation between teacher attachment and student growth. 47 propose a practical method for SIF downscaling. Guanter et al. [48] present a method based on RF for predicting diabetes. Subasi et al. [49] propose a decision support system for the diagnosis of migraine by RF. Classification of the driver's stress level is the main goal of [50]. Ayata et al. [51] propose an emotion recognition algorithm from multimodal physiological signals by using the random forest as one of the machine learning methods for recognition.

### 2.4. Industrial and Network fields

Zeraatpisheh et al. [52] use typical RF for producing the feature map in the industrial field. Du et al. [53] propose a rapid and accurate detection technique for pesticide detection by RF to construct a quantitative detection model. Improving the performance of mapping for mineral is the main goal of reference [54]. Liu et al. [55] propose an adaptive electrical period partition algorithm for open-circuit fault detection. Software fault prediction by ensemble techniques is investigated by [56]. In [57], the RF id is used to build a distributed energy system. A comprehensive image processing model is proposed by [58]. Ho et al. [59] uses RF to propose a framework that uses climate data to model hydropower generation. Zhou et al. [60] use RF for small and unbalanced datasets to create a risk prediction model for decision-making tool. Deng et al. [61] propose an authentication method for protecting high-value food products by RF. The forecast for agricultural products by RF is proposed by [62]. Jeong and Kim [63] use weighted random forest for the link prediction model. Khorshidpour et al. [64] present an approach to model an attack against classifiers with nondifferentiable decision boundary. Fusing multi-domain entropy and RF is the main goal of [65] for proposing a fault diagnosis method of the inter-shaft bearing. Analyzing the wine quality is presented by [66]. In the network field, Madhumathi and Suresh [67] develop a model to predict the future location of a dynamic sensor node in wireless communications. Fang et al. [68] propose an encrypted malicious traffic identification method. Detecting the intrusion in the network by typical RF is proposed by [69], and intrusion detection in the network security by tuning the RF parameter of the Moth-Flame optimization algorithm is presented by [70].

### 2.5. Physics, Text Processing, Tourism, and Urban Planning fields

In the physics field, Mingjing [71] measure and quantify the pH of soil by RF. 72 propose a model for extracting complex relationships between energy modulation and device efficiency. Zhang et al. [73] propose a model to accurately and effectively predict the UCS of LWSCC by a beetle antennae search algorithm for tuning the hyper-parameters of RF. The prediction of geotechnical parameters by typical RF is made by [74]. Creep index prediction by the RF algorithm to determine the optimal combination of variables is the main goal of [75]. In the text processing field, the comparison between RF and other classifiers is presented by [76] for finding the best classifiers in the subject literature of text classification. The random forest is used as one of the base learners of the ensemble model for fake news detection by [77]. Analyzing the reviewer's comment for sentiment analysis is the main goal of [78]. Zhang et al. [79] propose two novel label flipping attacks to evaluate the robustness of NB under noise by random forest. Recognizing newspaper text by RF is done by [80]. Madichetty and Sridevi [81] use RF as one of the classifiers for detecting the damage assessment tweets. Madasu and Elango [82] use the typical RF for feature selection for sentiment analysis. Chang et al. [83] use online customer reviews for opinion mining by RF. Text classification by simple RF is the goal of [84]. Onan and Toçouglu [85] present a method for document clustering and topic modeling on massive open online courses. Sentiment analysis of technical words in English by the Gini index for feature selection is done by [86]. Beck [87] uses ensemble learning and deep learning for sentiment classification scheme with high predictive performance in massive open online courses' reviews. Onan [88] present a deep learning based approach to sentiment analysis. This approach uses TF-IDF weighted Glove word embedding with CNN LSTM architecture. Onan and Tocoglu [89] present an effective sarcasm identification framework on social media data by pursuing the paradigms of neural language models and deep neural networks. In the tourism field, Rodriguez-Pardo et al. [90] propose a method based on simple RF for predicting the behaviour of tourists. Predicting the travel time to reduce traffic congestion is the main goal of [91]. Jamatia et al. 92 propose a method for tourist destinations' prediction. In urban planning, Baumeister et al. 93 rank the urban forest characteristics for cultural ecosystem services supply by typical RF. Forecasting road traffic conditions in done by [94]. The simulation of urban space development by RF is presented by [95]. Investigating the information on a gross domestic product for the analysis of economic development is presented by [96]. Mei et al. [97] propose a method to identify the spatiotemporal commuting patterns of the transportation system. In this brief review, the mentioned references are categorized in terms of innovation and functionality.

As can be seen from Table 1, RF has a high range of applications and variations in scope. In contrast, both in terms of quantity and quality, their innovations are often limited to set various parameters, and there is no significant innovation in the base learner combinations.

## 3. Learning Automata

**Figure 1:**
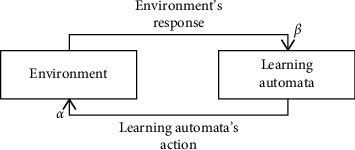
Interaction of learning automata with the environment.

Learning Automata (LA) is one of the learning algorithms that, after selecting different actions at different times, identify the best practices in terms of responses received from a random environment. LA selects an action from the set of actions in the vector of probabilities, and this action is evaluated in the environment. By using the received signal from the environment, the LA updates the probability vector and, by repeating this process, the optimal action is gradually identified. The classification problem can be formulated as a team of LA that operates collectively to optimize an objective function [102]. In Figure 1, the interaction of the learning automata and the environment is shown.

Finding the global optimum in the solution space is another advantage of using the LA. The LA can be formally represented by the quadruple

(8) $$\begin{array}{c} LA=\left\{\alpha ,\beta ,P,T\right\}, \end{array}$$

in which

(9) $$\begin{array}{c} \alpha =\left\{\alpha_{1},\alpha_{2},\ldots ,\alpha_{r}\right\} \end{array}$$

is the set of actions (outputs) of the LA; in other words, the set of inputs of the environment.

(10) $$\begin{array}{c} \beta =\left\{\beta_{1},\beta_{2},\ldots ,\beta_{r}\right\}, \end{array}$$

is the set of inputs of the LA; in other words, the set of outputs of the environment.

(11) $$\begin{array}{c} P=\left\{p_{1},p_{2},\ldots ,p_{r}\right\}, \end{array}$$

is the probability vector of the LA actions and

(12) $$\begin{array}{c} P\left(n+1\right)=T\left[P\left(n\right),\alpha \left(n\right),\beta \left(n\right)\right], \end{array}$$

is the learning algorithm.

In LA, three different models can be defined in the environment. In the P-Model, the environment presents the values of 0 or 1 as the output. In the Q-Model, the output values of the environment are discrete numbers between 0 and 1. In the S-Model, the output of the environment is the continuous value between 0 and 1. The selected actions by the LA are updated by both the signal received from the environment and using reward and penalty functions. The amount of allocated reward and penalty to the LA action can be defined in four ways: LRP, where the number of rewards and penalties are considered the same; LR*ε*P in which the amount of penalty is several times smaller than the reward; LRI in which the penalty amount is considered 0; and LIP, where the reward amount is considered 0 [103].

At each instant *n*, the action probability vector pi(n) is updated by the linear learning algorithm given in equation (13) if the chosen action ai(k) is rewarded by the environment, and it is updated according to equation (14) if the chosen action is penalized [104].

(13) $$\begin{array}{c} \left\{\begin{array}{c} p_{i}\left(n+1\right)=p_{i}\left(n\right)+a\left[1-p_{i}\left(n\right)\right],\\ p_{j}\left(n+1\right)=\left(1-a\right)p_{j}\left(n\right),\;\forall \;j,\;j\neq i, \end{array}\right. \end{array}$$

(14) $$\begin{array}{c} \left\{\begin{array}{c} p_{j}\left(n+1\right)=\left(1-b\right)p_{i}\left(n\right),\\ p_{j}\left(n+1\right)=\frac{b}{r-1}+\left(1-b\right)p_{j}\left(n\right),\;\forall \;j\;;\;j\neq i, \end{array}\right. \end{array}$$,

where “*a*” is the reward parameter, “*b*” is the penalty parameter, and “*r*” is the number of actions. The authors applied the LA in the proposed method, because:

- The LA presents an acceptable performance in uncertain situations.
- The LA does search action in the probability space.
- The LA requires simple feedback from the environment to optimize its state.
- Since the LA has a simple structure, it has a simpler implementation in both software and hardware.
- The LA is not constrained to use accuracy criteria for optimization usage.
- The LA is applicable in real-time usage since the LA is not involved with light computational complexity [105].

## 4. Proposed Method

The random forest is one of the methods of ensemble learning that all constructor classifiers are same type (i.e., decision tree). Therefore, the random forest is a homogeneous ensemble learning method. In this article, we intend to use the idea of reinforcement learning to increase the efficiency of random forest and add the ability to adapt to the conditions of the problem for this data mining algorithm. The details of the proposed method are described below.

The method proposed in this paper is based on the idea of reinforcement learning, and it employs the learning automata to implement the idea. The learning automata is the core of the proposed method, and by receiving feedback from the environment for each action, it updates the probability selection of the actions. In the proposed method, each base learner, all of which are decision tree, are considered as learning automata actions.

**Figure 2:**
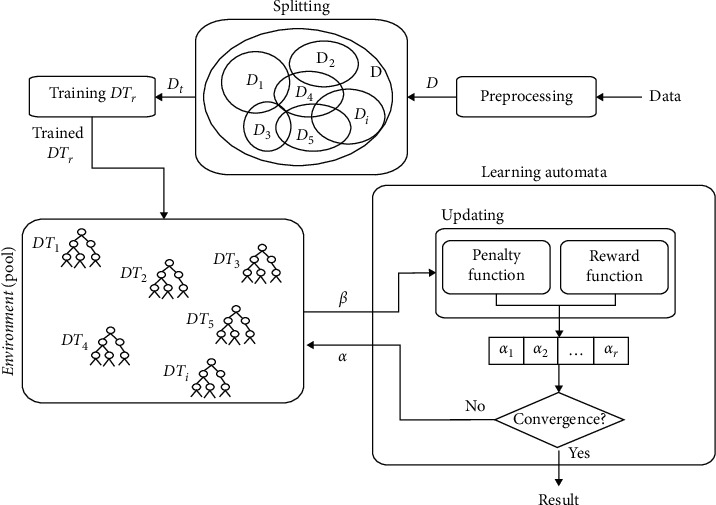
The block diagram of the proposed method.

In the proposed method, the training data are first randomly divided into N sections. In this division, N corresponds to the number of trees we want to have in the forest. Unlike the random forest, in which the predictive model works by averaging or voting between trees, in the proposed method, the predictive model is created using learning automata, which forms the core of the algorithm. The block diagram of the proposed method is shown in Figure 2.

The preprocessing step in the proposed method is a general step, and based on what type of data the processing area is dealing with, the details of this phase are determined. In the proposed method, at first, similar to the random forest method, the training data are divided into the number of base learners and randomly injected into the base learners. The difference between this step and the similar step in the random forest is that all the features in the feature vector are given to all base learners, and the feature replacement option is removed.

After the first run, the prediction models are created in the base learners and placed in a pool that is actually an interactive environment with the learning automata. The results obtained from the base learners for each new sample are given in the form of a reinforcement signal to the learning automata, which we know as the primary feedback of the environment. Depending on whether the received reinforcement signal is a reward or a penalty, the chances of selecting each of the base learners, -which they are the actions of the learning automata - are updated. It should be noted that the initial probability of selecting these actions is considered equal at the start. If we have *R* base learners to form the ensemble, the probability of the initial selection of each of them is equal to

(15) $$\begin{array}{c} p\left(DT_{r}\right)=\left(1/R\right). \end{array}$$

It is clear that the sum of the probabilities of all actions will be equal to 1.

(16) $$\begin{array}{c} \sum_{i=1}^{R}\left(pDT\right)=1. \end{array}$$

The initial probability of selecting actions is considered equal because all of them are homogeneous in terms of separating power.

In the proposed method, integration of the base learners is performed by the LA. Therefore, for each input in the test set, a linear LA is defined, and the action of each LA corresponds to selecting the base learners. The process of running base learners and receiving feedback from the environment continues until the probability of selecting actions converges to one of the base learners, or the number of repetitions for learning automata exceeds the predetermined limit. Once the probability of selections converges, then the result of the base learner, which has the highest probability of selection, is determined as the result of the ensemble for that particular input. In such a case, finding the global optimal is guaranteed by the algorithm, and because all the features in the feature vector are examined, rare modes are also covered, and the ability to adapt to the conditions of the problem space and independence from the domain is stabilized. In the proposed method, the random selection of subsets causes interdependence between trees. The depth of all the decision trees in the proposed method is considered equal. Each decision tree divides the training data differently at the leaf level. The pseudocode of the proposed method is shown in Algorithm 2.

In the learning automata block in Figure 2, there are two functions called the reward function and penalty function. Activation of one of these two functions is based on the type of reinforcement signal received from the environment. The received signal from the environment determines whether the result of the base learner activity or the selected action in the previous step was useful or not. If the result is useful, that action must be rewarded or, in other words, increase the probability of its selection. The increase in the probability of the selected action is determined by the parameters “a” and “b,” which are called the reward parameter and the penalty parameter, respectively.

To comply with (16), that is, the sum of the probabilities of all actions being equal to one, the probability of all other actions is reduced according to the size of the parameter “a.” If the result of the selected action is not useful, that action must also be penalized. In other words, the probability of that action must be reduced. To do this, the probability of selecting that action is reduced to the size of parameter “b,” and as a rewarding mode, and to observe (16), the probability of selecting other actions is increased by the size of the parameter “*b*.”

In the proposed method, the learning automata model environment is assumed to be the P-Model, where the environment defines zero and one values as outputs. Zero means reward, and one means penalty. If the correct answer is received from the selected base learner by the LA, the action of choice will be rewarded; otherwise, it will be penalized.

## 5. Evaluation

In order to thoroughly evaluate the efficiency of the proposed method, in this section, the details of the evaluation of the proposed method are presented separately from the data used and the experimental results.

### 5.1. Datasets

In order to evaluate the proposed method and to create an environment with the dynamic behaviour of data, different domains of applications have been selected. As mentioned in the previous sections, dynamic behaviour refers to the different results that an instance exhibits in different environmental conditions. Variety in the results of different environments is created by a specific domain. Text data are one of the most well-known types of data that exhibit such dynamic behaviour. In other words, these types of data are one of the optimal options for creating a dynamic environment, which proves the adaptability of the proposed method. The details of the selected data for the evaluation phase are shown in Table 2.

### 5.2. Experimental Result

**Figure 3:**
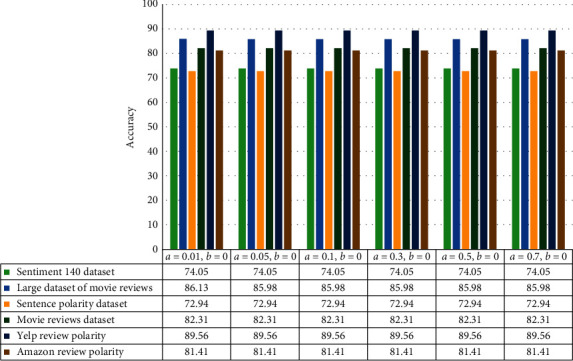
The results of the proposed method in LRI mode.

In order to evaluate the proposed method, eighteen datasets in different domains introduced in the previous section have been used. In the literature on learning automata, different modes have been considered for tuning learning automata; in this paper, three modes have been used to evaluate the proposed method. The LIP mode is not considered due to poor results. The evaluation results of each of the LRI, LRɛP, and LRP modes are shown in separate figures. In order to determine the optimal value for the reward and penalty parameters, six text datasets have been selected. The reason for this choice is the high diversity in the behaviour of textual data as well as a large number of samples and a large number of features of these six datasets. In the LRI mode, the value of the penalty parameter is considered to be zero, and the results of the proposed method in this mode are shown in Figure 3.

**Figure 4:**
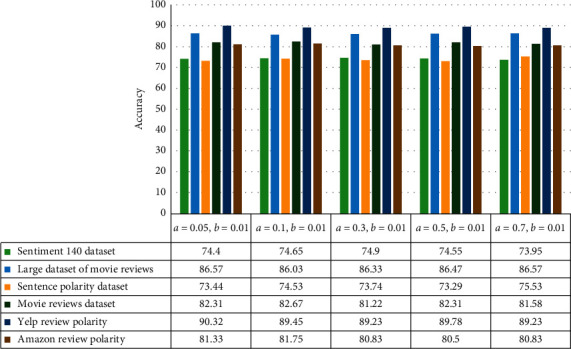
The results of the proposed method in the LRɛP mode.

Based on the literature on learning automata in the LRɛP mode, the value of the penalty parameter is considered to be much smaller than the value of the reward parameter. The results of the proposed method are shown in the LRɛP mode in Figure 4.

**Figure 5:**
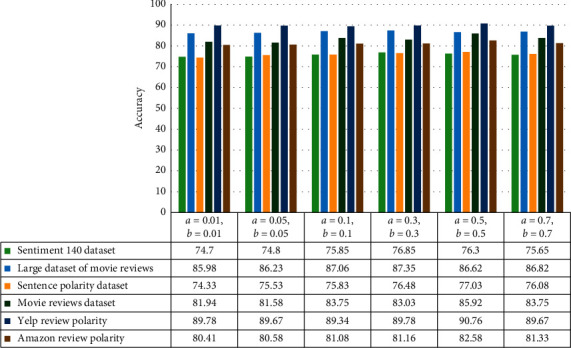
The results of the proposed method in the LRP mode.

As mentioned in the learning automata section, in the LRP mode, the values of the penalty and reward parameters are considered equal. The results of the proposed method in this mode are also shown in Figure 5.

A comparison of the results obtained from the implementation of the proposed method in three adjustable modes for learning automata shows that the settings on the LRP mode have resulted in the highest accuracy for identification. Then there are LRɛP and LRI modes. In the LRɛP mode, the setting *a* = 0.01, *b* = 0.01 is not considered, because these values are equal to the first values set in the LRP mode, and in order to prevent duplication of results in different tables, these settings have been removed from the LRɛP mode. For this reason, the number of experiments performed on LRɛP mode evaluations is one less than the other two. Considering that the settings of reward and penalty parameters in the LRP mode with the values of *a* = 0.5, *b* = 0.5 have resulted in the highest efficiency, evaluation has been done on other datasets with these settings. A comparison of the proposed method and similar approaches in the subject literature is shown in Table 3.

As can be seen in Table 3 from the point of view of accuracy, the proposed method offers better performance than the methods available in the subject literature, which indicates an improvement in the aggregation model of the base learners. This improvement is due to the use of reinforcement learning ideas of the method of aggregation of basic classifiers, which is known as base learner. The use of reinforcement learning ideas has improved the ability of the created ensemble, and it improved the ability to address issues in which data exhibit dynamic behaviour. The results of experiments performed on different data confirm the capabilities added to the random forest by the proposed method. As mentioned earlier, in the field of opinion mining, the type of text data is the most obvious data that exhibit such dynamic behaviour. Therefore, the optimal values for the reward and penalty parameters have been determined in these types of data, and these settings have been used for other types of data.

In addition to the accuracy criterion, other statistical criteria have been examined to evaluate the proposed method. As can be seen in Table 4, the proposed method has shown better results in both positive and negative classes than the methods available in the literature. Among the statistical criteria, Precision (P) determines the exactness of the results obtained from the classifier, and Recall (*R*) determines the completeness of the results obtained from the classifier. The results obtained from the test in the mentioned statistical criteria show that the proposed method has a high performance.

## 6. Discussion

In this section, more details of the proposed method are explained along with the reasons for the need to address these details. These include the details of the preprocessing step, tuning the learning automata parameters, as well as ranking the set of these parameters based on their performance.

### 6.1. Preprocessing

As explained in the proposed method section, the preprocessing step is a general step. In order for the evaluation, different data from different domains were examined. The preprocessing of textual data, along with the relevant details, is described below. It should be noted that preprocessing for other types of data, such as feature extraction, feature selection, normalization, noise removal, and other related preprocessing, has not been performed because all of them are taken as clean data from the UCI Repository [109]. And their basis for accuracy is based on previous research works that have used these data.

**Figure 6:**
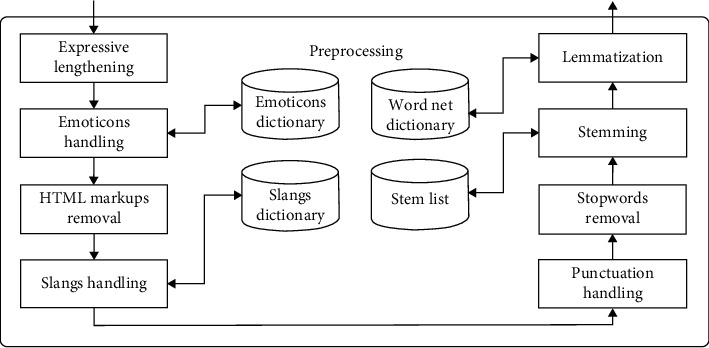
Details of the preprocessing step for text data.

In order to prepare textual data for the main process, the opinion mining domain is selected and the related preprocessing is as follows. The details of the preprocessing step for text data in opinion mining are shown in Figure 6.

 
*Expressive Lengthening*. Word lengthening or word stretching refers to the words that are elongated to express a particular emotion strongly, and the words with wrong spellings are corrected and replaced with their original words.
 
*Emoticons Handling.* It refers to the emoticons mentioned in the text that are replaced with their meaning, which makes it easier to analyze the emoticons.
 
*HTML Markups Removal.* HTML markups presented in the text are removed as they do not have any sentimental value attached to it.
 
*Slangs Handling.* The slangs are used for writing a given word, in short syllables, which depict the same meaning but save the time of typing. In slangs handling, the slangs presented in the text are replaced with their original words.
 
*Punctuation Handling.* Punctuations are used in a text to separate sentences and their elements, and to clarify their meaning. At punctuation handling, once the apostrophes are handled, all the remaining punctuations and numbers are removed.
 
*Stopwords Removal.* Stopwords do not carry much meaning and have no importance in the text. Stopwords are removed to get a simplified text.
 
*Stemming.* It refers to finding out the root or stem of a word. Removing various suffixes to reduce the number of words is the purpose of stemming.
 
*Lemmatization.* It returns the base or dictionary form of a word, which is known as the lemma. It is very similar to stemming, but it is more akin to synonym replacement.
 
*BoW creation.* The bag of word creation is the latest preprocess that is performed on the text preparation.

### 6.2. Tuning the Parameters of Reward and Penalty

In the subject literature of the learning automata, three different modes have been defined to tune the parameters of reward and penalty. In the proposed method, in which the idea of reinforcement learning is implemented using learning automata, all three adjustable modes of the parameters of reward and penalty are examined. The results of these three modes were presented in the experimental result section. In this paper, Friedman test statistical verification is used to determine which mode and which settings are best adjustable for the reward and penalty parameters. The values set for parameters “*a*” and “*b*” are shown in Table 5. Determining the numerical value of these parameters is based on the subject literature of learning automata. Of course, a wide variety of values can be considered for these two parameters. In this paper, an attempt has been made to tune the parameters in such a way that all the modes are considered so that they can be used to prove the efficiency of the proposed method compared to the previous methods.

### 6.3. Ranking

Friedman test statistical verification [110] is a ranking method that, the difference between the ranks assigned to each of the input samples, determines the optimal level of each option. In this paper, this verification method has been used to determine the optimal value of reward and penalty parameters as well as to compare the proposed method with the conventional methods in the subject literature of ensemble learning. The results are shown in Table 6.

As can be seen in Table 6, there is a significant difference between the rankings of the proposed method and the rankings of the traditional methods, which indicate an improvement in the efficiency of the proposed method compared to other methods. Among the three modes considered for tuning reward and penalty parameters, it is observed that the rankings have increased in LRI, LReP, and LRP modes, respectively. In the LRP mode, where the values of the reward and penalty parameters are considered the same, the highest efficiency is also observed. There is a significant difference between the Mean Rank of the best set of the reward and penalty parameters in the proposed method and this rank in the random forest method. The difference between the ranks is proof that the proposed method is optimal versus the traditional methods of aggregating classifiers to achieve a strong classification method.

### 6.4. Checking Convergence Rate

**Figure 7:**
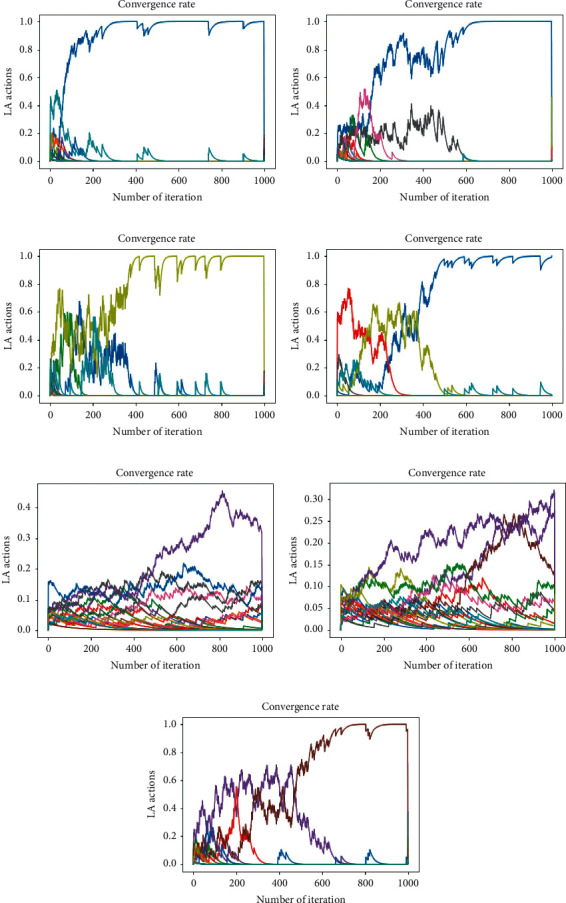
Convergence rate for different reward and penalty parameters. (a) *a* = 0.5, *b* = 0.5; (*b*) *a* = 0.3, *b* = 0.3; (c) a = 0.7, *b* = 0; (d) *a* = 0.1, *b* = 0.1; (e) *a* = 0.01, *b* = 0; (f) *a* = 0.05, *b* = 0.05; (g) *a* = 0.3, *b* = 0.

To more accurately address the proposed method in terms of efficiency, LA convergence has been investigated.  Figure 7 shows the convergence of LA actions for different amounts of reward and penalty variables. In most of the different settings for these two parameters, the convergence rate is high, and convergence to one of the actions usually occurs before reaching a certain number of iterations. As shown in Table 5, convergence at a lower rate occurred in some of the other settings that scored lower on the Friedman test.

### 6.5. Noise Resistance

**Figure 8:**
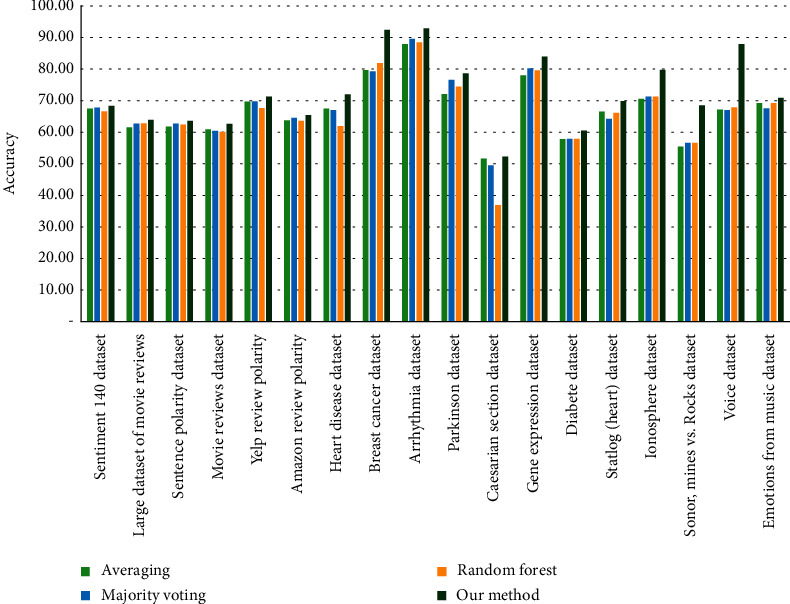
The evaluation of the proposed method in the presence of noise.

In order to more accurately evaluate the proposed method and determine the resistance of the proposed method to noise, another evaluation has been performed on the data presented in the previous section. This evaluation was performed by injecting 20% noise into clean data. The results of the evaluation on noisy data show that the proposed method, due to the use of learning automata, has high adaptability to the problem conditions, and in the presence of noise, contrary to conventional methods in the literature, the proposed method does not suffer a sharp decline, and in such conditions, it shows high efficiency compared to traditional methods. The evaluation of the proposed method in the presence of noise is shown in Figure 8.

## 7. Conclusion and Future Work

Base learner aggregation in ensemble learning should be done in such a way that the following points are met. First point: selecting a base learner leads to the highest performance achievable in the current situation. Second point: if the situation changes due to the dynamics of the problem, the structure of the ensemble will change in such a way that it has the greatest amount of compatibility with the conditions of the new environment. Therefore, in order to meet the above points and achieve an ensemble that is able to adapt to the dynamic conditions of the problem, in this paper, a new method based on the idea of reinforcement learning is proposed to integrate the base learners in the random forest. In the proposed method, learning automata is used to receive feedback from the environment and perform actions on it. The general procedure is to receive feedback from the environment, where the environment is a set of base learners that we intend to combine to achieve a better performance than individual base learners. Learning automata actions include choosing one of the base learners as the best base learner. The choice of action is based on receiving feedback from the environment. This causes the dynamic behaviour of data to be covered by using the idea of reinforcement learning. On the other hand, given that at each stage, learning automata strives to achieve the highest amount of achievable rewards, it is guaranteed to find the global optima in the problem space. Adaptability is another advantage of the proposed method compared to similar methods in the subject literature.

Due to the fact that in each step learning automata operates based on environmental conditions and received feedback from the environment, the ability to adapt to the problem is met. The results of the evaluations performed in different data show that the proposed method has the ability to achieve all the desired items mentioned above. Despite the fact that, unlike the random forest mechanism, all features are injected into all base learners in the proposed method, the efficiency of the proposed method in dealing with large-volume data has not decreased, and the results are more favorable than the classical methods. The proposed method is independent of the data type and has the ability to handle any other type of data in any field. In order to substantiate this claim, and in order to evaluate the proposed method, different types of data have been chosen. However, there are no restrictions on the proposed method for dealing with different types of data. In this paper, a new method for aggregating the base learners of the random forest using learning automata is proposed. Determining the optimal value for the parameters of reward and penalty in the form of self-tuning is one of the future works that the authors intend to do.

## Figures and Tables

**Algorithm 1:**
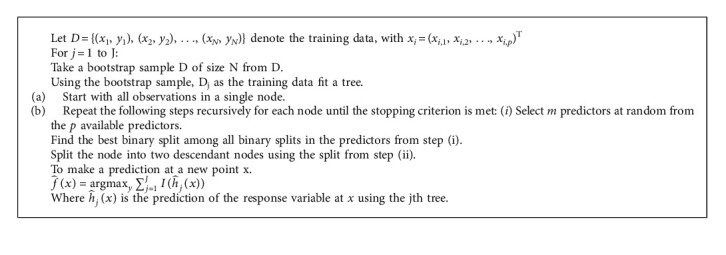
The random forest pseudocode for classification applications [1].

**Algorithm 2:**
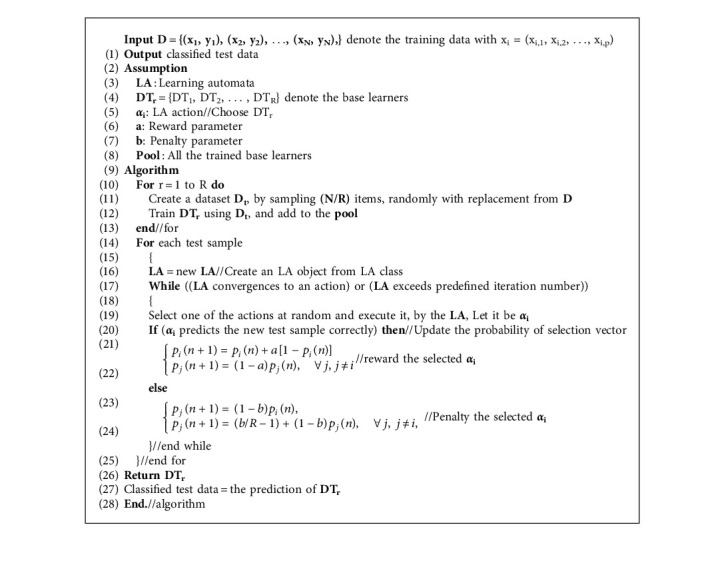
The pseudocode of the proposed method.

**Table 1:** Brief review of RF literature on functionality and innovation.

Type	Field	Paper
Functionality	Astronomy	[7], [8]
	Bioinformatics	[9], [10]
	Economics	[11], [12], [13]
	Global problem	[26], [27], [28]
	Healthcare	[32], [33], [34], [35], [36], [41], [98], [37], [39], [40], [42], [43], [45], [46], [47], [48], [49], [50], [51],
	Industrial	[52], [53], [54], [55], [56], [57], [58], [59], [60], [61], [62]
	Network	[63], [67], [68], [69], [99], [100]
	Physics	[71], [72]
	Text processing	[76], [77], [78], 80 [81], [82], [83], [84]
	Tourism	[91], [92]
	Urban planning	[93], [94], [95], [96], [97]
Innovative method	Economics	[14], [15]
	General	[16], [17], [18], [19], [101], [21], [22], [23], [24], [25]
	Global problem	[30], [31]
	Healthcare	[44]
	Industrial	[65]
	Network	[64]
	Physics	[73], [75]
	Text processing	[79], [86]

**Table 2:** Details of textual data used for evaluation.

Domain	Name	# Feature	# Instance
Text	Stanford—Sentiment 140 corpus [106]	Bag of word	1600000
	Large dataset of movie reviews [107]	Bag of word	50000
	Sentence polarity dataset v1.0 [108]	Bag of word	10662
	Internet movie database [105]	Bag of word	1400
	Yelp review [105]	Bag of word	598000
	Amazon review [105]	Bag of word	1000000
Healthcare	Heart disease dataset [105]	13	200
	Breast cancer dataset [105]	30	569
	Arrhythmia dataset [105]	279	454
	Parkinson dataset [105]	45	241
	Caesarean section dataset [105]	5	81
	Gene expression dataset [105]	255	801
	Diabetes dataset [105]	7	765
	Statlog (heart) dataset [105]	13	271
Physical	Ionosphere dataset [105]	34	352
	Sonar, mines vs. rocks dataset [105]	60	208
Sound	Voice dataset [105]	20	3168
	Emotions from music dataset [105]	28	592

**Table 3:** Comparison of the proposed method with similar approaches in the subject literature.

	Dataset	Averaging	Majority Voting	Random Forest	Our Method
Text	Sentiment140 dataset	74.54	75.50	74.30	**76.30**
	Large dataset of movie reviews	86.28	86.86	86.42	**86.62**
	Sentence polarity dataset	73.75	74.63	73.38	**77.03**
	Movie reviews dataset	81.58	81.58	81.67	**85.92**
	Yelp review polarity	89.47	90.32	89.74	**90.76**
	Amazon review polarity	80.86	81.66	80.97	**82.58**
Healthcare	Heart disease dataset	58.00	57.50	57.50	**65.00**
	Breast cancer dataset	97.41	97.36	96.49	**98.24**
	Arrhythmia dataset	80.71	85.71	81.31	**85.71**
	Parkinson dataset	63.95	64.58	64.58	**68.75**
	Caesarean section dataset	60.31	62.50	43.75	**68.75**
	Gene expression dataset	95.59	95.62	96.27	**98.75**
	Diabetes dataset	75.77	75.32	74.67	**76.62**
	Statlog (heart) data set	81.20	81.48	79.62	**85.18**
Physical	Ionosphere dataset	91.05	91.54	92.95	**95.77**
	Sonar, mines vs. rocks dataset	85.23	85.71	73.80	**88.09**
Sound	Voice dataset	76.38	76.18	76.49	**88.95**
	Emotions from music dataset	78.23	78.15	82.35	**84.03**

**Table 4:** Comparison of statistical criteria.

	Positive class			Negative class				Positive class			Negative class		
Method	P (%)	R (%)	F1 (%)	P (%)	R (%)	F1 (%)	Method	P (%)	R (%)	F1 (%)	P (%)	R (%)	F1 (%)
*Sentiment140 dataset*							Parkinson dataset						
MV	72.44	70.35	71.38	70.90	72.96	71.92	MV	69.23	66.67	67.92	**59.09**	61.9	60.47
RF	72.44	74.58	73.49	76.46	74.43	75.43	RF	69.23	66.67	67.92	**59.09**	61.9	60.47
OM	**75.20**	**80.36**	**77.69**	**81.17**	**76.16**	**78.59**	OM	**76.92**	**68.97**	**72.73**	**59.09**	**68.42**	**63.41**
**Large dataset of movie reviews**							**Caesarean section data set**						
MV	87.50	87.33	87.41	87.41	87.59	87.50	MV	55.56	71.43	62.5	**71.43**	55.56	62.5
RF	85.83	76.16	80.70	73.37	83.93	78.29	RF	33.33	50	40	57.14	40	47.06
OM	**87.80**	**87.97**	**87.88**	**88.10**	**87.93**	**88.01**	OM	**66.67**	**75**	**70.59**	**71.43**	**62.5**	**66.67**
**Sentence polarity dataset**							**Gene expression dataset**						
MV	75.63	72.83	74.20	72.80	75.61	74.18	MV	92.31	94.12	93.2	97.25	96.36	96.8
RF	74.62	67.78	71.08	65.95	72.94	69.27	RF	94.23	93.23	94.23	97.25	97.25	97.25
OM	**76.04**	**73.29**	**74.64**	**73.29**	**76.04**	**76.64**	OM	**98.08**	**98.08**	**98.08**	**99.08**	**99.08**	**99.08**
**Movie reviews dataset**							**Diabetes dataset**						
MV	83.10	82.52	82.81	81.84	82.09	81.78	MV	90.29	76.86	83.04	45.1	69.7	54.76
RF	77.46	71.43	74.32	76.41	73.98	70.54	RF	90.29	76.23	82.67	43.14	68.75	53.01
OM	**84.51**	**83.33**	**83.92**	**82.22**	**83.46**	**82.84**	OM	**91.26**	**77.69**	**83.93**	**47.07**	**72.73**	**57.14**
**Yelp review polarity**							**Voice dataset**						
MV	**90.85**	88.04	88.42	87.11	**90.11**	88.59	MV	64.58	76.09	69.86	84.85	76.24	80.31
RF	80.64	85.94	83.21	86.22	81.00	83.53	RF	64.58	76.75	70.14	85.4	76.35	80.62
OM	90.00	**89.43**	**89.71**	**88.89**	89.49	**89.19**	OM	87.82	86.55	87.18	89.81	90.81	90.3
**Amazon review polarity**							**Emotions from music dataset**						
MV	82.68	79.45	81.03	**79.38**	82.62	80.97	MV	83.58	78.87	81.16	71.15	77.08	74
RF	79.12	74.56	76.77	73.98	87.61	76.22	RF	88.06	**81.94**	84.89	**75**	82.98	78.79
OM	**83.70**	**79.52**	**81.56**	79.21	**83.45**	**81.28**	OM	**92.54**	81.58	**86.71**	73.08	**88.37**	**80**
**Heart disease dataset**							**Sonar, mines vs. Rocks dataset**						
MV	**61.90**	59.09	60.47	52.63	55.56	54.05	MV	**87.5**	**87.5**	**87.5**	**83.33**	**83.33**	**83.33**
RF	**61.90**	59.09	60.47	52.63	55.56	54.05	RF	79.17	76	77.55	66.67	70.59	68.57
OM	**61.90**	**68.42**	**65.00**	**68.42**	**61.90**	**65.00**	OM	**87.5**	**87.5**	**87.5**	**83.33**	**83.33**	**83.33**
**Breast cancer data set**							**Statlog (heart) data set**						
MV	95.74	97.83	96.77	**98.51**	97.06	97.78	MV	**79.19**	79.19	79.19	83.33	83.33	83.33
RF	**97.87**	93.88	95.83	95.52	98.46	96.97	RF	75	78.26	76.6	83.33	80.65	81.67
OM	**97.87**	**97.87**	**97.87**	**98.51**	**98.51**	**98.51**	OM	**79.19**	**86.36**	**82.61**	**90**	**84.38**	**87.1**
**Arrhythmia data set**							**Ionosphere data set**						
MV	88.37	**82.61**	85.39	**83.33**	88.89	86.02	MV	82.14	95.83	88.46	97.67	89.36	93.33
RF	83.72	78.26	80.9	79.17	79.74	73.85	RF	85.71	96	90.57	97.67	91.3	94.38
OM	**93.02**	80	**86.02**	79.19	**92.68**	**85.39**	OM	**89.29**	**100**	**94.34**	**100**	**93.48**	**96.63**

P, *R*, and F1 refer to Precision, Recall, and F1-score. MV: majority voting, RF: random forest, and OM: our method.

**Table 5:** Numerical values tuned for reward and penalty parameters.

Mode	Parameter						
LRI	*a*	0	0.1	0.1	0.3	0.5	0.7
	*b*	0	0	0	0	0	0
LR*ε*P	*a*	0.1	0.1	0.3	0.5	0.7	
	*b*	0	0	0	0	0	
LRP	*a*	0	0.1	0.1	0.3	0.5	0.7
	*b*	0	0.1	0.1	0.3	0.5	0.7

**Table 6:** Friedman test statistical verification results for ranking the parameters of reward and penalty and comparing the proposed method with the literature.

Method	Tuning	Mean rank	Final rank
LRP	*a* = 0.5, *b* = 0.5	19.17	1
LRP	*a* = 0.3, *b* = 0.3	16.83	2
LRP	*a* = 0.7, *b* = 0.7	15.58	3
MV	Majority voting	14.67	4
LRP	*a* = 0.1, *b* = 0.1	13.92	5
LReP	*a* = 0.05, *b* = 0.01	12.17	6
LReP	*a* = 0.1, *b* = 0.01	11.83	7
LReP	*a* = 0.5, *b* = 0.01	10.08	8
LRP	*a* = 0.05, *b* = 0.05	9.58	9
RF	Random forest	9.17	10
LRP	*a* = 0.01, *b* = 0.01	8.75	11
LIR	*a* = 0.01, *b* = 0	8.42	12
LIR	*a* = 0.05, *b* = 0	7.67	13
LIR	*a* = 0.1, *b* = 0	7.67	13
LIR	*a* = 0.3, *b* = 0	7.67	13
LIR	*a* = 0.5, *b* = 0	7.67	13
LIR	*a* = 0.7, *b* = 0	7.67	13
AV	Averaging	7.58	14
LReP	*a* = 0.3, *b* = 0.01	7.17	15
LReP	*a* = 0.7, *b* = 0.01	6.75	16

## Data Availability

The authors declare that all the data are available publicly at the UCI repository.
